# Biomass Cooking Fuel Use and Screen-Detected Hypertension Among Peruvian Women Without Known Hypertension: A National Cross-Sectional Study

**DOI:** 10.3390/life16081293

**Published:** 2026-08-06

**Authors:** Víctor Juan Vera-Ponce, Jhosmer Ballena-Caicedo, Marcos García-Rodríguez, Witre Omar Padilla, José Celso Paredes Carranza, Fiorella E. Zuzunaga-Montoya

**Affiliations:** Facultad de Medicina (FAMED), Universidad Nacional Toribio Rodríguez de Mendoza de Ama-zonas (UNTRM), Chachapoyas 01001, Peru; 7330178022@untrm.edu.pe (J.B.-C.); marcos.garcia@untrm.edu.pe (M.G.-R.); witre.padilla@untrm.edu.pe (W.O.P.); jose.paredes@untrm.edu.pe (J.C.P.C.); fiorella.zuzunaga@untrm.edu.pe (F.E.Z.-M.)

**Keywords:** indoor air pollution, biomass, hypertension, women, cross-sectional studies, Peru, health surveys

## Abstract

Household biomass combustion remains a source of household air pollution and unequal exposure among women. Its association with hypertension is biologically plausible, but evidence from Latin America remains limited, particularly when the analysis is restricted to women without a prior hypertension diagnosis, which represents the population of greatest interest for early screening. This study aimed to estimate the association between biomass cooking fuel use and screen-detected hypertension among Peruvian women aged 15–49 years without known hypertension. We conducted an analytical cross-sectional study using pooled DHS 2014–2024 microdata. Nonpregnant women aged 15–49 years with valid blood pressure measurements, cooking fuel information, complete covariates, and no self-reported diagnosis or current antihypertensive treatment were included. Screen-detected hypertension was defined as mean systolic blood pressure ≥ 140 mmHg or mean diastolic blood pressure ≥ 90 mmHg. Poisson regression with robust variance based on the complex sampling design was used to estimate prevalence ratios (PRs). The main model was adjusted for age, altitude, area of residence, natural region, education, wealth quintile, and survey year. Sensitivity, fuel-specific, effect-modification, positivity, sequential-adjustment, extended-adjustment, and continuous blood-pressure analyses were performed. The analytical sample included 301,575 women, of whom 78,499 used biomass/solid fuel and 18,571 had screen-detected hypertension. The weighted prevalence of screen-detected hypertension was 8.5% among clean-fuel users and 6.4% among biomass/solid-fuel users. The crude association was inverse (crude PR: 0.75; 95% CI: 0.71–0.79), but it reversed after adjustment (adjusted PR: 1.20; 95% CI: 1.10–1.31). Wood was associated with a higher adjusted prevalence (adjusted PR: 1.25; 95% CI: 1.14–1.36). Coal/charcoal showed an imprecise null estimate (adjusted PR: 1.00; 95% CI: 0.76–1.31). The association was stronger in urban than in rural areas (adjusted PR: 1.28 vs. 1.09; interaction *p* = 0.005). Among Peruvian women without known hypertension, biomass/solid cooking fuel use was associated with a higher adjusted prevalence of screen-detected hypertension, supporting the inclusion of clean-fuel transition within integrated cardiovascular prevention strategies.

## 1. Introduction

Household air pollution remains a health and equity challenge because exposure is concentrated in populations that rely on polluting fuels to cook or heat the home. Globally, approximately 2 billion people still use polluting fuels or technologies at home, and incomplete combustion of biomass, coal, or other solid fuels generates high concentrations of fine particulate matter and other respirable pollutants in indoor environments [1,2,3]. Although the energy transition has advanced, access to clean cooking remains unequal, particularly in rural areas, lower socioeconomic households, and settings with limited energy infrastructure [3].

The cardiovascular relevance of this exposure should be understood against the backdrop of elevated blood pressure as one of the leading risk factors for global health loss, with hypertension continuing to affect a substantial proportion of adults [4,5]. Epidemiological and mechanistic evidence has linked household air pollution to systemic inflammation, oxidative stress, endothelial dysfunction, autonomic alterations, and increased risk of cardiovascular events, although the magnitude of associations varies by outcome, exposure assessment method, and population context [2,6].

For blood pressure and hypertension (HTN) specifically, the available evidence remains heterogeneous. Observational studies in low- and middle-income countries have described associations between solid fuel use and elevated blood pressure, particularly among women exposed during cooking activities, but they have also shown variation by age, urbanicity, socioeconomic status, and fuel type [7,8]. In addition, interventions involving improved cookstoves or transitions to clean fuels have not consistently shown clear blood pressure reductions over short follow-up periods, indicating that interpretation must consider exposure intensity, duration, adherence, ventilation, residual exposure, and baseline household differences [9].

In Peru, this issue is particularly relevant because cooking fuel use varies substantially across territorial and social contexts. Dependence on solid fuels has been associated with rural residence, poverty, altitude, and environmental characteristics, and studies conducted in high Andean settings have documented high household exposures in homes that cook with biomass [10,11]. Local trials and studies have contributed to characterizing exposure and the cardiopulmonary effects of clean cooking interventions, but their findings do not replace the need for national population-based analyses that simultaneously account for the sampling design, the unequal distribution of exposure, and comparability across groups [12,13]. Therefore, we aimed to evaluate the association between biomass or solid cooking fuel use and hypertension detected by objective survey measurement among Peruvian women.

## 2. Materials and Methods

### 2.1. Study Design

We conducted an observational, analytical cross-sectional study based on secondary microdata from the Peruvian DHS. The manuscript was written and organized according to the STROBE recommendations for observational studies [14]. Compliance with STROBE items is summarized in Table S1.

### 2.2. Data Source

The DHS is a household-based population survey conducted continuously by the Instituto Nacional de Estadística e Informática del Perú (INEI). Its databases, methodological documentation, and national and departmental results are publicly available through the INEI institutional portal [15].

The survey uses a probabilistic, stratified, multistage design with selection of clusters and households and provides sampling weights for representative estimates according to the domains defined by the survey. Data used in this study came from the DHS modules on household characteristics, health, blood pressure, history of hypertension, medication use, sociodemographic characteristics, and sampling design [15].

### 2.3. Study Population

The source population comprised women aged 15 to 49 years included in the DHS 2014–2024 rounds. The primary analytical population included nonpregnant women with valid systolic and diastolic blood pressure measurements, valid information on cooking fuel, positive sampling weight, stratum and primary sampling unit identifiers, and complete covariate data. We excluded women with known hypertension at the time of the survey, defined as a report of previous hypertension diagnosis or current antihypertensive medication use. Therefore, current antihypertensive medication use was not treated as a baseline pharmacological characteristic in the primary analytical sample because it was absent by design.

The decision to restrict the primary analysis to women without known hypertension was based on outcome classification: a woman who has been diagnosed and treated may have normal blood pressure during field measurement. Consequently, including medication use in the outcome after excluding women with known hypertension would have been conceptually inconsistent with the target population of the primary analysis.

### 2.4. Outcomes

The primary outcome was screen-detected hypertension, defined as mean systolic blood pressure ≥ 140 mmHg or mean diastolic blood pressure ≥ 90 mmHg, consistent with epidemiological thresholds used to classify hypertension in adults [16]. This definition was applied only within the population without known hypertension and was based on objective blood pressure measurements recorded by the DHS.

Systolic and diastolic blood pressure were calculated from available measurements considered valid in the database. Values outside physiologically plausible ranges defined during data cleaning were treated as missing: systolic blood pressure <60 or >260 mmHg and diastolic blood pressure <30 or >160 mmHg. Measurement validity was based on the complete-measurement indicator available in the DHS and the presence of nonmissing systolic and diastolic blood pressure values [15].

For secondary analyses, two additional outcomes were defined. Total hypertension was defined as measured elevated blood pressure, a self-reported previous hypertension diagnosis, or self-reported current antihypertensive medication use. Measured elevated blood pressure was defined using the same systolic or diastolic threshold, regardless of previous diagnosis or medication use. Complete operational definitions are presented in Table S2.

### 2.5. Exposure

The primary exposure was the type of fuel used for cooking, obtained from the DHS household fuel variable [15]. Use of electricity, liquefied petroleum gas, natural gas, or biogas was classified as clean fuel. Use of coal or lignite, charcoal, wood, straw, shrubs, grass, agricultural crop residues, or dung was classified as biomass/solid fuel.

The main comparison was biomass/solid fuel versus clean fuel. Secondary analyses separated specific solid fuel categories: wood, coal/charcoal, and other solid fuels. Responses corresponding to kerosene, other fuel, not cooking at home, no response, or missing fuel were excluded from the main classification because they did not consistently represent the contrast of interest between clean fuels and biomass/solid fuels.

### 2.6. Covariates

**Figure 1:**
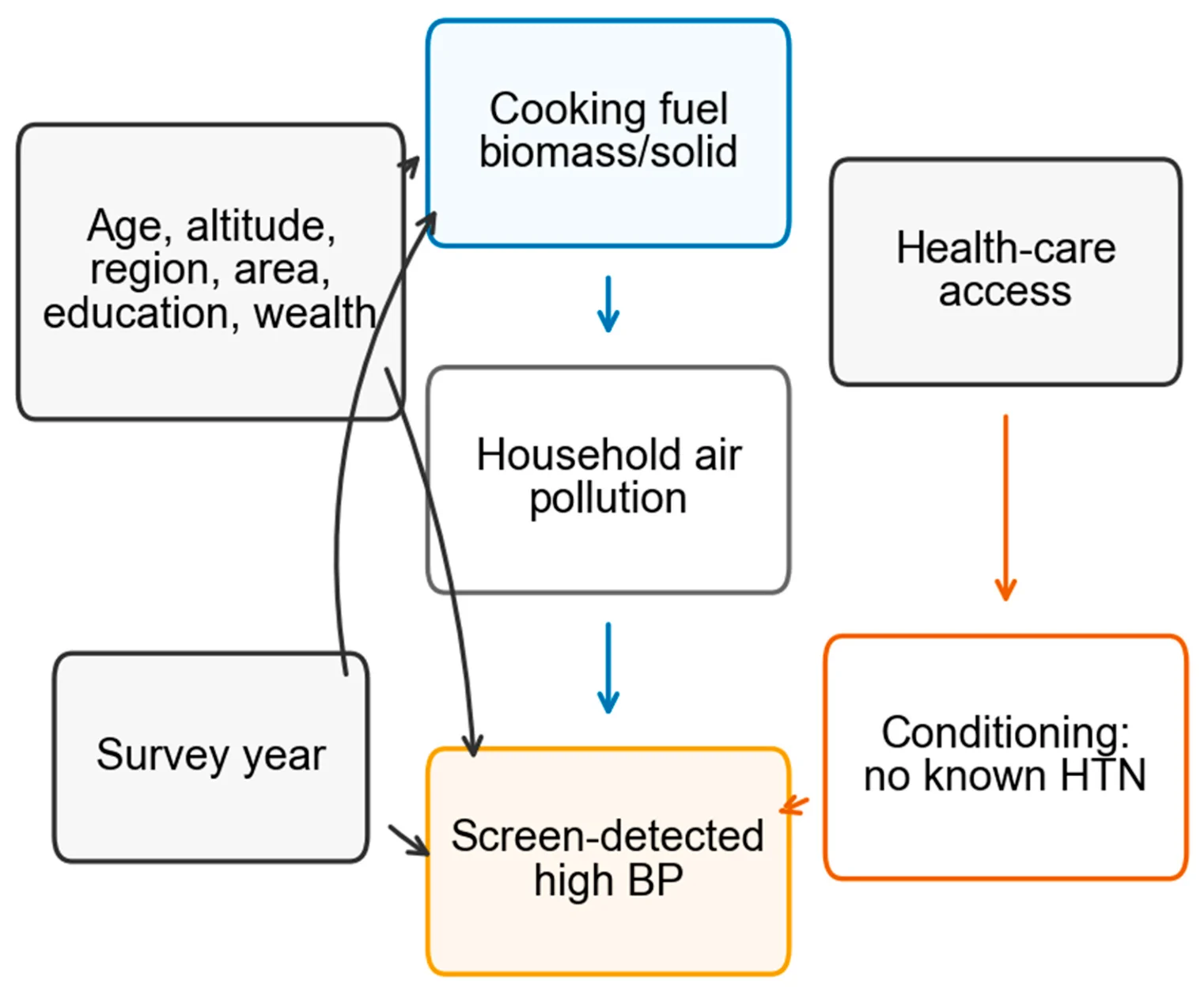
Conceptual directed acyclic graph (DAG). Arrows indicate the assumed causal direction between variables in the hypothesized relationship between biomass/solid cooking fuel and screen-detected high blood pressure (BP). BP: blood pressure; HTN: hypertension; DAG: directed acyclic graph.

Adjustment covariates were selected a priori based on their expected relationship with cooking fuel use, blood pressure, or differential access to diagnosis. The main model included age, cluster altitude, area of residence, natural region, educational level, wealth quintile, and survey year. All variables were obtained from DHS modules and dictionaries [15]. The conceptual DAG used to organize the relationship among exposure, covariates, and outcome is shown in Figure 1.

Age and altitude were modeled as continuous variables using restricted cubic splines with four knots to avoid imposing a linear association with the outcome. Area of residence was classified as urban or rural; natural region as Metropolitan Lima, rest of the coast, highlands, or jungle; educational level as no education, primary, secondary, or higher education; and wealth index as ordinal quintiles provided by the DHS.

### 2.7. Statistical Analysis

All analyses incorporated the complex sampling design of the DHS using weights, strata, and primary sampling units. When combining annual rounds, stratum and cluster identifiers were made unique by survey year to preserve the design structure in the pooled file. Strata with a single primary sampling unit were handled by variance centering.

Characteristics of the analytical population were summarized using unweighted absolute frequencies and weighted percentages, means, or standard deviations, as appropriate. Absolute standardized differences were used to compare the composition of fuel groups because descriptive *p* values tend to be uninformative in large samples.

The main association was estimated using Poisson regression with a log link and robust variance based on the survey design, reporting prevalence ratios and 95% confidence intervals. This approach was selected because the outcome was prevalent and the prevalence ratio is more interpretable than the odds ratio in a cross-sectional analysis. The adjusted model included biomass/solid fuel as the main exposure and controlled for age, altitude, area, natural region, education, wealth, and DHS year.

To characterize the reversal between crude and adjusted estimates, we fitted sequential adjustment models and leave-one-block-out models. These analyses were used descriptively to identify covariate blocks contributing most to confounding; because geographic and socioeconomic variables were strongly correlated, they were not interpreted as a unique decomposition of causal confounding.

To complement the dichotomous screen-detected hypertension outcome, systolic and diastolic blood pressure were analyzed as continuous outcomes using survey-weighted linear regression with robust variance and the same covariate set as the main model. Estimates were reported as adjusted mean differences in mmHg.

Positivity and common support were evaluated using a propensity score for biomass/solid fuel use estimated with weighted logistic regression and the same covariates as in the main model. This analysis did not replace the main model; it was used as a diagnostic of comparability between exposed and unexposed women and as the basis for a sensitivity analysis restricted to common support.

Sensitivity analyses were performed to evaluate the stability of the results under contestable methodological decisions: analysis in the full sample using the total hypertension definition, analysis in the full sample using measured blood pressure only, exclusion of medicated women only, exclusion of previous diagnosis only, restriction to women aged 18 to 49 years, inclusion of pregnant women, exclusion of Metropolitan Lima, and restriction to common support of the propensity score. Specific solid fuels were also evaluated against clean fuel.

An extended adjustment sensitivity model additionally included BMI, employment status, and current smoking status when these variables were available and harmonized across survey rounds. This model was interpreted as a robustness analysis rather than as a replacement for the prespecified main model.

Effect modification was explored using interaction terms between biomass/solid fuel and area of residence, natural region, altitude category, age group, DHS period, and wealth quintile. Interaction tests were based on design-adjusted Wald tests, and stratified results were interpreted considering the size of exposed strata and positivity diagnostics.

Missing data were not imputed. The main analysis used complete cases for exposure, outcome, covariates, weight, and design variables. Data cleaning and models were run in StataNow 19; tables, figures, and the reproducible document were generated using reproducible scripts.

### 2.8. Ethical Considerations

The study used only publicly available, anonymized secondary microdata released by INEI. We did not access personal identifiers or contact participants. Because this was a secondary analysis of anonymized data, with no intervention, recontact, or primary data collection, review by an institutional ethics committee was not required for this analysis. This decision is consistent with ethical considerations for secondary data analyses and with the public and anonymized nature of DHS datasets [15,17].

## 3. Results

### 3.1. Sample Selection

The pooled DHS 2014–2024 file included 398,744 records for the study period. After restricting the population to women aged 15 to 49 years, 355,588 records remained. We excluded pregnant women, observations without valid blood pressure measurement, observations without valid clean fuel or biomass/solid fuel information, women with known hypertension, and observations with incomplete covariates, sampling design, or weight. The primary analytical sample comprised 301,575 women without a previous hypertension diagnosis or reported antihypertensive medication use, as shown in Figure S1.

### 3.2. Characteristics of the Analytical Population

In the main sample, 223,076 women used clean fuel and 78,499 used biomass or solid fuel. The weighted mean age was 30.6 years among both clean fuel users and biomass/solid fuel users. Biomass/solid fuel users were concentrated in rural areas, the highlands, and the poorest wealth quintile; the weighted rural percentage was 73.5% in the biomass/solid fuel group and 7.4% in the clean fuel group. The weighted mean altitude was 2079.9 m among biomass/solid fuel users and 707.6 m among clean fuel users. Absolute standardized differences were larger for area of residence, wealth, altitude, natural region, and education, indicating compositional differences between exposure groups. Full characteristics are shown in Table 1.

Anthropometric profiles also differed by cooking fuel type. Mean BMI was 26.7 kg/m^2^ among clean-fuel users and 25.6 kg/m^2^ among biomass/solid-fuel users, with an absolute standardized difference of 0.242.

The weighted mean systolic blood pressure was 116.8 mmHg among clean fuel users and 115.9 mmHg among biomass/solid fuel users. The weighted mean diastolic blood pressure was 71.7 mmHg and 70.8 mmHg, respectively. The weighted prevalence of screen-detected hypertension was 8.5% in the clean fuel group and 6.4% in the biomass/solid fuel group before covariate adjustment.

The comparison between included and excluded women is presented in Table S3, and the characteristics of women with known HTN versus those without known HTN are shown in Table S4.

### 3.3. Main Association Between Biomass/Solid Fuel Use and Screen-Detected HTN

In crude analysis, biomass/solid fuel use was associated with a lower prevalence of screen-detected hypertension compared with clean fuel (crude PR: 0.75; 95% CI: 0.71 to 0.79). After adjustment for age, altitude, area of residence, natural region, education, wealth, and survey year, the association changed direction and biomass/solid fuel use was associated with a higher prevalence of screen-detected hypertension (adjusted PR: 1.20; 95% CI: 1.10 to 1.31). These results are presented in Table 2.

In the fuel-specific analysis, wood was associated with a higher adjusted prevalence of screen-detected hypertension compared with clean fuel (adjusted PR: 1.25; 95% CI: 1.14 to 1.36). For coal/charcoal, the adjusted PR was 1.00 (95% CI: 0.76 to 1.31), and for other solid fuels it was 0.92 (95% CI: 0.72 to 1.17). Table S7 shows these specific contrasts.

Sequential adjustment models showed that the crude inverse association was progressively attenuated after adjustment for altitude and changed direction after adding area of residence. Additional adjustment for natural region and wealth further increased the estimate toward the fully adjusted PR. In leave-one-block-out models, removing wealth produced the largest attenuation of the full-model estimate, followed by area of residence and natural region. These findings indicate that the crude-to-adjusted reversal was mainly explained by geographic and socioeconomic confounding rather than by a protective association of biomass/solid fuel use. Complete results are shown in Table S10.

### 3.4. Common Support and Positivity Diagnostics

**Figure 2:**
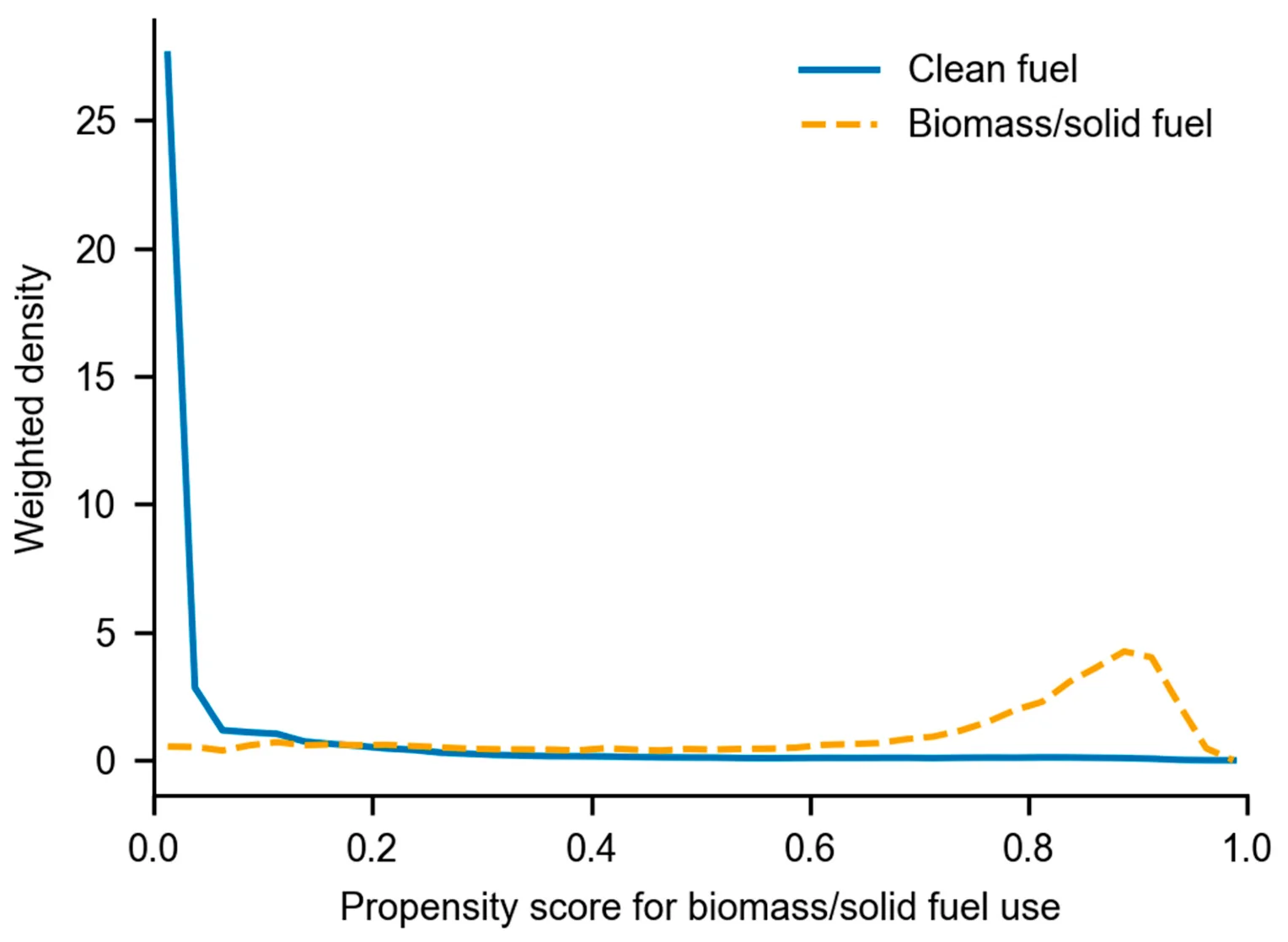
Distribution of the propensity score for biomass/solid fuel use.

The propensity score distribution showed separation between exposure groups, with a higher concentration of clean fuel users at low score values and biomass/solid fuel users at high score values. Common support ranged from 0.0001 to 0.9648. Outside common support accounted for 13,154 of 223,076 clean fuel users and 95 of 78,499 biomass/solid fuel users. Figure 2 presents the propensity score distribution, and Table S9 summarizes positivity diagnostics. Sparse strata were observed in exposure cells by region, area, and wealth, particularly in the richest quintile, where only 7 women were exposed to biomass/solid fuel.

### 3.5. Temporal Trends

**Figure 3:**
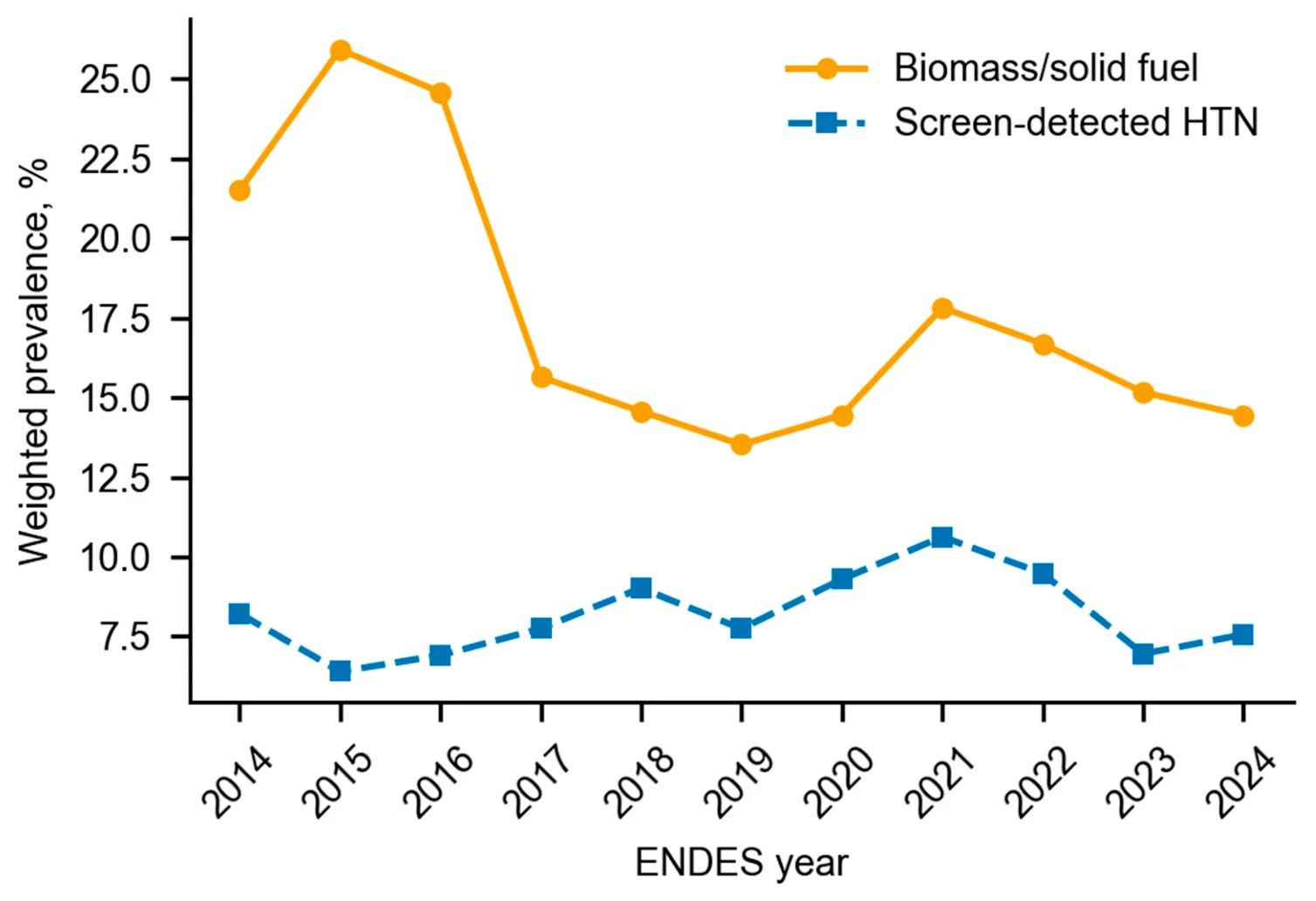
Temporal trend in biomass/solid fuel use and screen-detected hypertension (HTN) among Peruvian women without known hypertension, according to ENDES year. ENDES: Encuesta Demográfica y de Salud Familiar; HTN: hypertension.

The weighted prevalence of biomass/solid fuel use varied over the study period, with higher values at the beginning of the period and lower values after 2017. The weighted prevalence of screen-detected hypertension showed annual variation, with a maximum value in 2021 within the analytical population. These trends are presented in Figure 3.

### 3.6. Sensitivity Analyses

Sensitivity analyses yielded adjusted estimates similar to the main analysis. In the full sample using the total hypertension definition, the adjusted PR was 1.18 (95% CI: 1.11 to 1.25). In the full sample using only measured blood pressure, the adjusted PR was 1.18 (95% CI: 1.09 to 1.27). When only medicated women were excluded, the adjusted PR was 1.20 (95% CI: 1.10 to 1.31). Among women aged 18 years or older, the adjusted PR was 1.22 (95% CI: 1.12 to 1.33). When pregnant women were included, the adjusted PR was 1.19 (95% CI: 1.09 to 1.30), and when Metropolitan Lima was excluded, it was 1.19 (95% CI: 1.09 to 1.30). In the analysis restricted to common support of the propensity score, the adjusted PR was 1.20 (95% CI: 1.10 to 1.32). An extended adjustment sensitivity model additionally including BMI, employment status, and current smoking status yielded an adjusted PR of 1.23 (95% CI: 1.12 to 1.34; *p* < 0.001; n = 293,404; events = 18,045). Complete results are shown in Table S6; selected sensitivity analyses are summarized graphically in Figure S2.

In complementary continuous-outcome analyses, biomass/solid fuel use was associated with small lower adjusted mean systolic and diastolic blood pressure values compared with clean fuel users: −0.85 mmHg (95% CI: −1.08 to −0.61) for systolic blood pressure and −0.96 mmHg (95% CI: −1.11 to −0.80) for diastolic blood pressure. Fuel-specific estimates are shown in Table S11.

### 3.7. Adjusted Model and Spline Functions

The main model included age and altitude using restricted cubic splines. In the fully adjusted model, biomass/solid fuel use retained an adjusted PR of 1.20 (95% CI: 1.10 to 1.31). Table S5 presents the full model terms, and Figure S3 shows the estimated functional form for age and altitude.

### 3.8. Effect Modification

In stratified analyses, the adjusted association between biomass/solid fuel and screen-detected hypertension was stronger in urban areas (adjusted PR: 1.28; 95% CI: 1.14 to 1.43) than in rural areas (adjusted PR: 1.09; 95% CI: 0.96 to 1.24), with an interaction *p* value of 0.005. By natural region, the association was stronger in the rest of the coast (adjusted PR: 1.39; 95% CI: 1.20 to 1.62), with an interaction *p* value of 0.015. There was no statistical evidence of modification by altitude or age group. By DHS period, the adjusted PR was 1.40 (95% CI: 1.19 to 1.64) in 2014–2017, 1.00 (95% CI: 0.86 to 1.17) in 2018–2020, and 1.12 (95% CI: 0.97 to 1.29) in 2021–2024, with an interaction *p* value < 0.001. By wealth, estimates were unstable in higher strata because exposed women were scarce, especially in the richest quintile. Full results are presented in Table S8. Figure S4 shows the weighted regional distribution of biomass/solid fuel use and screen-detected hypertension.

## 4. Discussion

### 4.1. Main Findings

In this national analysis of Peruvian women aged 15 to 49 years without known hypertension, use of biomass or solid fuel for cooking was associated with a higher adjusted prevalence of hypertension detected by objective survey measurement. The crude association was lower among biomass/solid fuel users but reversed direction after adjustment, reflecting marked compositional differences between exposure groups, particularly by rurality, region, altitude, education, and wealth. Sequential and leave-one-block-out analyses confirmed that this reversal was driven mainly by geographic and socioeconomic covariate blocks. The continuous blood-pressure analyses did not show higher adjusted mean systolic or diastolic blood pressure among biomass/solid-fuel users, indicating that the dichotomous association should be interpreted as a threshold-based finding rather than as evidence of a uniform upward shift in average blood pressure. Results were consistent in sensitivity analyses that modified the outcome definition, restricted the age range, included pregnant women, excluded Metropolitan Lima, or limited the sample to common support of the propensity score.

### 4.2. Comparison with Other Studies

Our findings are compatible with multinational studies that have evaluated solid fuels and blood pressure in women of reproductive age. Arku et al. analyzed Demographic and Health Surveys from 10 countries, including Peru, and reported associations between solid fuel use and hypertension or elevated blood pressure among premenopausal women [7]. Unlike that multinational approach, our study focused on a single country, used 11 consecutive DHS rounds, and restricted the primary outcome to screen-detected hypertension among women without previous diagnosis or treatment, allowing the estimand to be interpreted as unknown hypertension detected through population-based screening.

The observed association is also related to prior Peruvian evidence from high-exposure settings. In a high Andean population, Burroughs Peña et al. reported a higher probability of prehypertension and hypertension among people with daily exposure to biomass smoke [13]. Our analysis did not measure personal particulate matter exposure and did not focus inference on a specific geographic area, but it complements that evidence by showing that the association persists in a weighted national sample, even after adjustment for altitude and natural region.

Findings should also be interpreted alongside evidence from clean cooking interventions, which have not always shown rapid blood pressure changes. In the CHAP trial conducted in Puno, the liquefied petroleum gas intervention reduced exposure to household air pollution but did not show clear differences in blood pressure or other cardiopulmonary outcomes over 1 year of follow-up [12]. This apparent difference does not necessarily contradict our results because a population-based cross-sectional study estimates the association between usual fuel conditions and measured blood pressure, whereas an intervention trial evaluates changes after technology substitution under a specific period, adherence, and exposure history.

The heterogeneity by area, region, and period observed in our study is consistent with literature describing solid fuel dependence as a socially structured phenomenon. In Peru, solid fuel dependence has been linked to environmental and socioeconomic conditions, and international analyses have shown that solid fuel use among women follows unequal patterns by country, time, and household characteristics [8,10]. Therefore, comparing biomass users and nonusers requires more than conventional statistical adjustment; it also requires evaluation of positivity and common support because exposure is concentrated in specific territorial and socioeconomic profiles.

The fuel-specific results should also be interpreted cautiously. The absence of a positive adjusted association for coal/charcoal should not be interpreted as evidence that these fuels are harmless. Coal/charcoal users represented a much smaller exposed subgroup than wood users, and the confidence interval was wide. In addition, the DHS fuel variable captures the main cooking fuel rather than emissions intensity, stove type, ventilation, cooking duration, or mixed fuel use. Therefore, the contrast between wood and coal/charcoal may reflect limited precision and contextual patterns of fuel use rather than differences in toxic potential alone.

### 4.3. Implications for Public Health

From a national perspective, these results suggest that the transition to clean fuels should be considered within an integrated cardiovascular prevention agenda, not only as an environmental or respiratory intervention. Unknown hypertension is relevant because it identifies a fraction of women with elevated blood pressure who do not report diagnosis or treatment. In settings where preventive care is unequal, population surveys with objective measurement can help identify populations in which environmental exposure and cardiovascular underdiagnosis overlap.

Cardiovascular prevention in exposed populations should not be reduced to household energy policy alone. Nutritional and selected nutraceutical or functional-food approaches have been discussed as adjunctive strategies in cardiovascular risk management, particularly for metabolic risk profiles [18]. However, these approaches do not remove household air-pollution exposure and should be interpreted as complementary to, rather than substitutes for, clean-fuel transition, blood-pressure screening, and equitable access to care.

Internationally, this study provides evidence from a middle-income country with substantial geographic heterogeneity, persistent rurality, and incomplete energy transition. This combination is common in Latin America, Asia, and Africa, where clean cooking policy intersects with energy poverty, gender, health-care access, and prevention of noncommunicable diseases. WHO policy tools for clean household energy and international frameworks for universal clean cooking access emphasize that interventions should be monitored using indicators of adoption, sustained use, and health benefits [19,20].

Our findings also have implications for how evidence is communicated. Reporting total hypertension prevalence is not enough when part of the population has already been diagnosed or is receiving treatment. In survey studies, distinguishing total hypertension from hypertension detected by objective measurement helps avoid incompatible interpretations and makes the estimand more transparent. This distinction is especially important when access to diagnosis and treatment depends on education, wealth, urbanicity, or proximity to the health system.

### 4.4. Implications for Policy and International Research

For public policy, the evidence supports an intersectoral interpretation: cooking fuel is not merely a household choice but a marker of infrastructure, energy poverty, domestic workload, environmental exposure, and unequal access to health. The potential benefits of clean cooking include health, time, productivity, gender equity, and climate; therefore, programs require coordination across health, energy, environment, social development, and local government sectors [20,21].

For international research, this study illustrates the usefulness of repeated analyses of national surveys when specific estimands are formulated. Restricting to women without known hypertension is not intended to estimate the total burden of hypertension but rather a form of hypertension detected through population-based screening. This approach may be replicable in other demographic and health surveys that collect blood pressure, cooking fuel, previous diagnosis, and treatment data.

Future research should combine population surveillance with more direct exposure measurement. Fuel variables provide a practical proxy, but they do not capture ventilation, cooking time, concurrent use of multiple fuels, stove efficiency, or personal pollutant concentrations. Longitudinal studies, PM2.5 or black carbon measurements, and implementation evaluations may clarify which combinations of energy transition, adherence, and exposure reduction are needed to observe sustained cardiovascular benefits.

### 4.5. Limitations

This study has limitations. Its cross-sectional design precludes establishing temporality or causality, and reverse causation cannot be ruled out; for example, a previous diagnosis, symptoms, or household health changes could affect fuel decisions, although the primary analysis excluded women with known hypertension. Exposure was based on the main fuel reported for cooking and not on personal pollutant measurements or detailed information on ventilation, cooking duration, or mixed fuel use. Blood pressure was measured during the survey and is not equivalent to a clinical diagnosis confirmed in repeated visits. Previous diagnosis and medication use were self-reported, so misclassification may occur. In addition, although we adjusted for relevant covariates and evaluated common support, residual confounding remains possible, particularly from diet, physical activity, salt intake, health-care access, outdoor ambient air pollution, housing characteristics, and other factors unavailable or not measured homogeneously in the database. Restricting to women aged 15 to 49 years without known hypertension improves estimand coherence but limits generalizability to men, older women, pregnant women, and people with diagnosed or treated hypertension.

## 5. Conclusions

Among Peruvian women aged 15 to 49 years without known hypertension, biomass or solid cooking fuel use was associated with a higher adjusted prevalence of hypertension detected by objective measurement in the DHS 2014–2024. The association was robust to several sensitivity analyses and to extended adjustment for BMI, employment status, and current smoking status, although complementary continuous blood-pressure models did not show higher adjusted mean systolic or diastolic blood pressure among biomass/solid-fuel users. The concentration of exposure among rural, poor women and residents of specific regions requires interpreting the results within the observed limits of comparability.

We recommend strengthening strategies for sustained transition to clean fuels in populations with high dependence on biomass, incorporating assessment of actual use, affordability, continuity of supply, and effective exposure reduction. In parallel, surveys and health programs should integrate blood pressure measurement, identification of unknown hypertension, and surveillance of household exposure, especially among women from rural and lower-socioeconomic settings. Future studies should use longitudinal designs, environmental or personal pollutant measurements, information on mixed fuel use, and analyses that explicitly distinguish total hypertension, known hypertension, and screen-detected hypertension.

## Figures and Tables

**Table 1:** Weighted characteristics of women without known hypertension by type of cooking fuel (BMI: body mass index; DHS: Demographic and Family Health Survey).

Variable	Total	Clean Fuel	Biomass/Solid Fuel	ASD
Age, years	30.6 (9.8)	30.6 (9.6)	30.6 (10.4)	0.003
BMI, kg/m^2^	26.5 (5.0)	26.7 (5.1)	25.6 (4.5)	0.242
Area of residence				
Urban	80.9	92.6	26.5	1.818
Rural	19.1	7.4	73.5	1.818
Natural region				
Metropolitan Lima	36.0	43.5	0.9	1.193
Rest of coast	26.6	29.0	15.6	0.328
Highlands	24.5	17.2	58.4	0.937
Jungle	12.9	10.2	25.1	0.399
Altitude, m	950.9 (1303.2)	707.6 (1134.0)	2079.9 (1436.1)	1.061
Education				
No education	1.4	0.5	5.3	0.288
Primary	15.2	9.5	41.5	0.787
Secondary	48.3	49.0	44.7	0.086
Higher education	35.2	40.9	8.5	0.812
Wealth quintile				
Poorest	16.9	4.5	74.1	2.026
Poor	20.7	20.3	22.2	0.046
Middle	21.6	25.6	3.3	0.666
Rich	21.5	26.0	0.4	0.819
Richest	19.4	23.5	0.0	0.784
DHS year				
2014	8.0	7.7	9.8	0.074
2015	9.5	8.6	13.9	0.17
2016	9.7	8.9	13.4	0.145
2017	9.6	9.8	8.5	0.048
2018	9.7	10.1	8.0	0.074
2019	9.5	10.0	7.3	0.098
2020	6.4	6.6	5.2	0.061
2021	9.6	9.6	9.7	0.002
2022	9.4	9.6	8.9	0.023
2023	9.4	9.7	8.1	0.058
2024	9.1	9.4	7.4	0.074
Systolic blood pressure, mmHg	116.6 (15.1)	116.8 (15.3)	115.9 (14.4)	0.057
Diastolic blood pressure, mmHg	71.6 (9.8)	71.7 (9.9)	70.8 (9.3)	0.099
Screen-detected hypertension				
No	91.9	91.5	93.6	0.081
Yes	8.1	8.5	6.4	0.081

Values are weighted percentages unless otherwise specified. Continuous variables are presented as weighted means with standard deviations. ASD: absolute standardized difference between groups. ASD greater than 0.1 indicates relevant imbalance. Current antihypertensive medication use is not shown because previous hypertension diagnosis or current antihypertensive treatment was an exclusion criterion for the primary analytical population.

**Table 2:** Association between biomass/solid fuel use and screen-detected HTN.

Exposure	Unweighted *n*	Weighted Prevalence of HTN, %	Crude PR (95% CI)	Adjusted PR (95% CI)
Clean fuel	223,076	8.5	1.00	1.00
Biomass/solid fuel	78,499	6.4	0.75 (0.71–0.79)	1.20 (1.10–1.31)
Wood	71,142	6.5	0.77 (0.72–0.82)	1.25 (1.14–1.36)
Coal/charcoal	2612	7.0	0.82 (0.62–1.07)	1.00 (0.76–1.31)
Other solid fuels	4745	3.8	0.44 (0.35–0.55)	0.92 (0.72–1.17)

HTN: hypertension; PR: prevalence ratio; CI: confidence interval. The crude model includes only fuel type. The adjusted model includes age, altitude, area of residence, natural region, education, wealth quintile, and survey year.

## Data Availability

The microdata used are publicly available through the Instituto Nacional de Estadística e Informática (INEI) repository: https://proyectos.inei.gob.pe/microdatos/ (accessed on 1 May 2026).

## Supplementary Materials

The following supporting information can be downloaded at: https://www.mdpi.com/article/10.3390/life16081293/s1, Table S1: STROBE checklist for cross-sectional studies; Table S2: Operational definitions; Figure S1: Flow diagram for analytical sample selection; Table S3: Comparison between included and excluded women; Table S4: Characteristics of women with known HTN versus women without known HTN; Table S5: Fully adjusted model for the main analysis; Table S6: Sensitivity analyses by outcome definition and analytical population; Table S7: Analysis by specific fuel type; Table S8: Effect modification; Table S9: Positivity diagnostics; Table S10: Sequential and leave-one-block-out adjustment models for the association between biomass/solid fuel use and screen-detected hypertension; Table S11: Adjusted mean differences in systolic and diastolic blood pressure by cooking fuel type; Figure S2: Forest plot of sensitivity analyses including the extended adjustment sensitivity model; Figure S3: Spline functions for age and altitude; Figure S4: Weighted regional prevalence of biomass/solid fuel use and screen-detected HTN.
