# Supplementary material for: Biomass Cooking Fuel Use and Screen-Detected Hypertension Among Peruvian Women Without Known Hypertension: A National Cross-Sectional Study
Source: Life (Basel). 2026 Aug 6;16(8):1293. doi: 10.3390/life16081293 (PMC13514650; doi:10.3390/life16081293)

Supplementary Figure S1. Flow diagram for analytical sample selection.

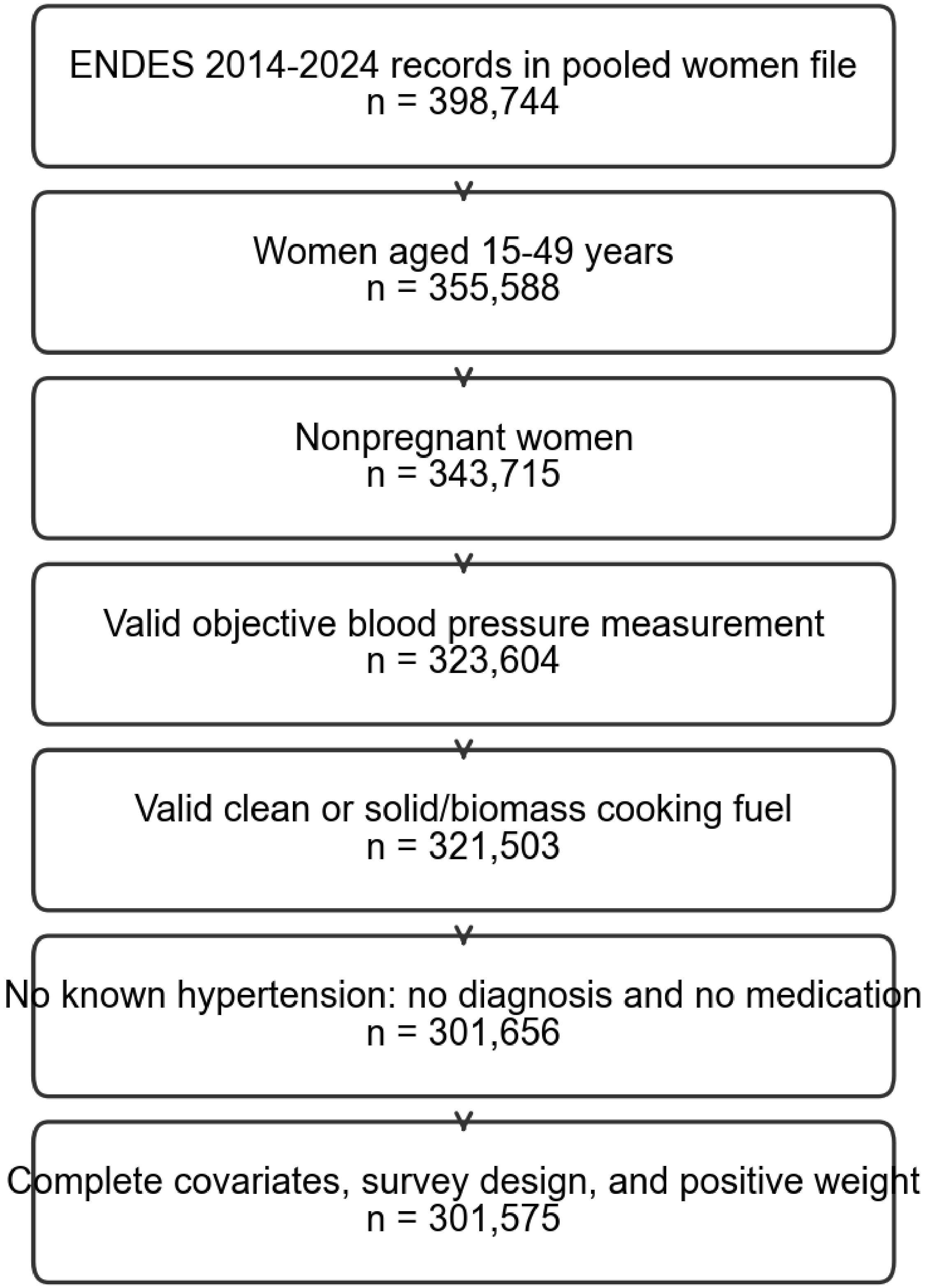

Supplement: Supplementary file 1 [file life-16-01293-s001.zip › BiomassHTN_SM_Figure_S1_Flow.pdf]
