# Supplementary material for: Biomass Cooking Fuel Use and Screen-Detected Hypertension Among Peruvian Women Without Known Hypertension: A National Cross-Sectional Study
Source: Life (Basel). 2026 Aug 6;16(8):1293. doi: 10.3390/life16081293 (PMC13514650; doi:10.3390/life16081293)
Supplement: Supplementary file 1 [file life-16-01293-s001.zip › BiomassHTN_SM_Table_S10_Sequential_LeaveOneOut_HIGHLIGHTED.pdf]

Supplementary Table S10. Sequential and leave-one-block-out adjustment models for the association between biomass/solid fuel use and screen-detected hypertension.

Panel A. Sequential adjustment.

Model	Adjustment set	n	Events	PR (95% CI)	p
Crude	None	301,575	18,571	0.75 (0.71-0.79)	<0.001
Model 1	Age + survey year	301,575	18,571	0.76 (0.72-0.81)	<0.001
Model 2	Model 1 + altitude	301,575	18,571	0.89 (0.84-0.94)	<0.001
Model 3	Model 2 + area of residence	301,575	18,571	1.03 (0.96-1.11)	0.360
Model 4	Model 3 + natural region	301,575	18,571	1.12 (1.04-1.21)	0.005
Model 5	Model 4 + education	301,575	18,571	1.13 (1.05-1.23)	0.002
Full model	Age + altitude + area + natural region + education + wealth + survey year	301,575	18,571	1.20 (1.10-1.31)	<0.001

Panel B. Leave-one-block-out from the full model.

Model	Adjustment set	n	Events	PR (95% CI)	p
Full model	None removed	301,575	18,571	1.20 (1.10-1.31)	<0.001
Full minus age	Age	301,575	18,571	1.20 (1.10-1.31)	<0.001
Full minus altitude	Altitude	301,575	18,571	1.21 (1.11-1.32)	<0.001
Full minus area	Area of residence	301,575	18,571	1.17 (1.07-1.27)	<0.001
Full minus natural region	Natural region	301,575	18,571	1.18 (1.09-1.29)	<0.001
Full minus education	Education	301,575	18,571	1.21 (1.11-1.32)	<0.001
Full minus wealth	Wealth quintile	301,575	18,571	1.13 (1.05-1.23)	0.002
Full minus survey year	Survey year	301,575	18,571	1.21 (1.11-1.32)	<0.001

PR: prevalence ratio; CI: confidence interval. Models used survey-weighted Poisson regression with robust variance and the complex survey design.
