# Supplementary material for: Biomass Cooking Fuel Use and Screen-Detected Hypertension Among Peruvian Women Without Known Hypertension: A National Cross-Sectional Study
Source: Life (Basel). 2026 Aug 6;16(8):1293. doi: 10.3390/life16081293 (PMC13514650; doi:10.3390/life16081293)
Supplement: Supplementary file 1 [file life-16-01293-s001.zip › BiomassHTN_SM_Table_S11_Continuous_BP_HIGHLIGHTED.pdf]

Supplementary Table S11. Adjusted mean differences in systolic and diastolic blood pressure by cooking fuel type.

Exposure	Unweighted n	SBP adjusted mean difference, mmHg (95% CI)	SBP p	DBP adjusted mean difference, mmHg (95% CI)	DBP p
Clean fuel	223,076	Reference		Reference	
Biomass/solid fuel	78,499	-0.85 (-1.08 to -0.61)	<0.001	-0.96 (-1.11 to -0.80)	<0.001
Wood	71,142	-0.75 (-1.00 to -0.51)	<0.001	-0.96 (-1.13 to -0.80)	<0.001
Coal/charcoal	2,612	-0.19 (-1.29 to 0.91)	0.736	-0.74 (-1.49 to 0.02)	0.057
Other solid fuels	4,745	-2.64 (-3.29 to -2.00)	<0.001	-1.01 (-1.45 to -0.58)	<0.001

SBP: systolic blood pressure; DBP: diastolic blood pressure; CI: confidence interval. Models used survey-weighted linear regression with robust variance and the same covariate set as the main model: age, altitude, area of residence, natural region, education, wealth quintile, and survey year.
