# Supplementary material for: Biomass Cooking Fuel Use and Screen-Detected Hypertension Among Peruvian Women Without Known Hypertension: A National Cross-Sectional Study
Source: Life (Basel). 2026 Aug 6;16(8):1293. doi: 10.3390/life16081293 (PMC13514650; doi:10.3390/life16081293)
Supplement: Supplementary file 1 [file life-16-01293-s001.zip › BiomassHTN_SM_Table_S1_STROBE_REVISED_HIGHLIGHTED.pdf]

Supplementary Table S1. STROBE checklist for cross-sectional studies.

Item	STROBE section	Requirement	Approach in this manuscript
1a	Title and abstract	Indicate the study design using a commonly used term in the title or abstract.	The title and abstract identify the study as a pooled analytical cross-sectional analysis of DHS 2014-2024.
1b	Title and abstract	Provide an informative and balanced abstract of what was done and found.	The abstract includes background, objective, methods, main results with PRs and 95% CIs, and conclusions focused on screen-detected hypertension.
2	Introduction	Explain the scientific background and rationale for the study.	The introduction describes household biomass air pollution, its cardiovascular relevance, the global burden of hypertension, and the need for population-based evidence in Peru and Latin America.
3	Introduction	State specific objectives, including any prespecified hypotheses where applicable.	The objective is stated at the end of the introduction: to evaluate the association between biomass/solid cooking fuel use and screen-detected hypertension among Peruvian women without known hypertension. No directional causal hypothesis is formulated.
4	Methods	Present the key elements of the study design early in the paper.	The design section describes an observational, analytical cross-sectional study based on pooled secondary DHS 2014-2024 microdata.
5	Methods	Describe the setting, locations, relevant dates, and data collection period.	The data source section identifies the Peruvian DHS, conducted by INEI, reports the 2014-2024 period, and describes the household population-based nature and use of public microdata.
6a	Methods	For cross-sectional studies, describe eligibility criteria, sources, and methods of participant selection.	The analytical population is defined as nonpregnant women aged 15 to 49 years with valid blood pressure,

			valid fuel information, complete covariates, positive weight, and available design variables; women with previous HTN diagnosis or antihypertensive medication use were excluded.
6b	Methods	For matched studies, describe matching criteria and the number of matched participants.	Not applicable. The study is cross-sectional and does not use individual matching or paired participant selection.
7	Methods	Clearly define outcomes, exposures, predictors, potential confounders, and effect modifiers.	Screen-detected hypertension, total hypertension, measured elevated blood pressure, biomass/solid fuel, clean fuel, specific fuel, age, altitude, area, natural region, education, wealth, DHS year, and assessed effect modifiers are defined. The revised manuscript also defines continuous SBP and DBP analyses and adds BMI, employment status, and smoking status for the extended adjustment sensitivity model.
8	Methods	For each variable of interest, indicate data sources and measurement methods; if more than one group is included, describe comparability of methods.	Blood pressure comes from objective DHS measurements; fuel, previous diagnosis, medication, and covariates come from DHS modules. Variables were measured with common survey procedures within each round and harmonized for the pooled analysis.
9	Methods	Describe efforts to address potential sources of bias.	The main analysis was restricted to women without known HTN to avoid treatment-related masking; sociodemographic and geographic covariates were adjusted for; common support, positivity, and sensitivity with alternative

			outcome definitions were evaluated.
10	Methods	Explain how the study size was determined.	No a priori sample size was calculated; all eligible records available in DHS 2014-2024 were included according to inclusion, exclusion, and data completeness criteria.
11	Methods	Explain how quantitative variables were handled in the analyses; describe groupings if used.	Age and altitude were modeled with restricted cubic splines; some variables were grouped for description or effect modification, such as age groups, altitude categories, and DHS periods. BMI was summarized as a continuous anthropometric variable in kg/m2, and SBP and DBP were additionally analyzed as continuous outcomes in mmHg.
12a	Methods	Describe all statistical methods, including those used to control for confounding.	Poisson models with log link, robust variance, and complex survey design were used to estimate prevalence ratios; the adjusted model included age, altitude, area, natural region, education, wealth, and DHS year. Additional reviewer-requested analyses included an extended adjustment sensitivity model, sequential adjustment models, leave-one-block-out models, and survey-weighted linear regression for continuous SBP and DBP.
12b	Methods	Describe methods used to examine subgroups and interactions.	Effect modification was evaluated using interaction terms between biomass/solid fuel and area, natural region, altitude, age, DHS period, and wealth; stratified estimates and interaction p values were reported.

12c	Methods	Explain how missing data were addressed.	The main analysis used complete cases for exposure, outcome, covariates, weights, and design variables; missing data were not imputed. Exclusions due to completeness are documented in the sample flow.
12d	Methods	For cohort studies, explain how loss to follow-up was addressed.	Not applicable. The design is cross-sectional and does not include longitudinal follow-up or losses during follow-up.
12e	Methods	Describe sensitivity analyses.	Analyses were performed in the full sample with total HTN, full sample with measured BP, exclusion of medicated women only, exclusion of diagnosis only, restriction to ages 18-49 years, inclusion of pregnant women, exclusion of Metropolitan Lima, and restriction to common support. The sensitivity analyses were expanded to include an extended adjustment model with BMI, employment status, and current smoking status.
13a	Results	Report the number of participants at each stage of the study.	The flow diagram presents DHS 2014-2024 records, women aged 15 to 49 years, nonpregnant women, women with valid blood pressure, valid fuel, no known HTN, complete covariates, and the final sample.
13b	Results	Provide reasons for non-participation at each stage, where applicable.	For secondary databases, individual field nonparticipation was not evaluated; the manuscript reports analytical exclusions due to pregnancy, blood pressure measurement, fuel, known HTN, and missing data.
13c	Results	Consider using a flow diagram.	A sample selection flow diagram is included as Figure S1.

14a	Results	Describe participant characteristics and distribution of exposures and potential confounders.	Table 1 presents weighted characteristics by fuel; Tables S3 and S4 compare included/excluded women and women with known HTN versus without known HTN.
14b	Results	Indicate the number of participants with missing data for each variable of interest.	The sample flow and included/excluded comparison document losses due to critical variables; the final analysis is restricted to complete covariates, exposure, outcome, and design variables.
14c	Results	For cohort studies, summarize follow-up time.	Not applicable. The study is cross-sectional and contains no individual temporal follow-up.
15	Results	For cross-sectional studies, report the number of outcome events or summary measures.	The number of women with screen-detected hypertension and the weighted prevalence by fuel group are reported; Table 2 presents events and prevalences.
16a	Results	Present unadjusted and adjusted estimates with precision; indicate which confounders were adjusted for and why.	Crude and adjusted PRs with 95% CIs are reported; adjustment included age, altitude, area, natural region, education, wealth, and DHS year because of their relationship with exposure, blood pressure, and diagnostic access.
16b	Results	Report category boundaries when continuous variables were categorized.	Categories are reported for age, altitude, DHS periods, wealth, natural region, and specific fuel; age and altitude were modeled primarily as continuous variables using splines.
16c	Results	When relevant, translate relative estimates into absolute risk measures.	Absolute weighted prevalences of screen-detected hypertension by fuel group are presented alongside prevalence ratios.
17	Results	Report other analyses performed, such as subgroup, interaction, and sensitivity analyses.	Analyses by specific fuel type, sensitivity by outcome definition and population, common support,

			effect modification, and temporal trends are reported. Reviewer-requested supplementary analyses now include sequential/leave-one-block-out adjustment models and continuous blood-pressure outcome models.
18	Discussion	Summarize key results with reference to study objectives.	The discussion begins with a main findings paragraph highlighting the adjusted association between biomass/solid fuel and screen-detected hypertension and consistency in sensitivity analyses.
19	Discussion	Discuss limitations, considering the direction and magnitude of potential bias.	Cross-sectional design, reverse causation, exposure measurement by reported fuel, absence of personal measurements, survey blood pressure, self-reported diagnosis/medication, and residual confounding are discussed.
20	Discussion	Provide a cautious interpretation of results, considering objectives, limitations, multiplicity, similar studies, and relevant evidence.	The interpretation avoids causal language, compares findings with multinational and Peruvian studies, distinguishes association from causality, and situates the results within the estimand of screen-detected hypertension among women without known HTN.
21	Discussion	Discuss generalizability or external validity.	The manuscript notes that findings apply to Peruvian women aged 15 to 49 years without known HTN and do not directly represent men, older women, pregnant women, or people with diagnosed/treated HTN.
22	Other information	Declare the funding source and role of funders.	The declarations section states that the study was self-funded; there were no external funders with a role

		in design, analysis, interpretation, or writing.

STROBE = Strengthening the Reporting of Observational Studies in Epidemiology.
