# Supplementary material for: Biomass Cooking Fuel Use and Screen-Detected Hypertension Among Peruvian Women Without Known Hypertension: A National Cross-Sectional Study
Source: Life (Basel). 2026 Aug 6;16(8):1293. doi: 10.3390/life16081293 (PMC13514650; doi:10.3390/life16081293)
Supplement: Supplementary file 1 [file life-16-01293-s001.zip › BiomassHTN_SM_Table_S2_Definitions_REVISED_HIGHLIGHTED.pdf]

Supplementary Table S2. Operational definitions.

Element	Operational definition
Primary analytical population	Women aged 15 to 49 years, nonpregnant, with valid BP, valid fuel, complete covariates/design/weight, and no previous HTN diagnosis or report of antihypertensive medication use.
Screen-detected HTN	Mean SBP >=140 mmHg or mean DBP >=90 mmHg among women without known HTN.
Total HTN	Mean SBP >=140 mmHg, mean DBP >=90 mmHg, previous HTN diagnosis, or report of medication use.
Measured elevated blood pressure	Mean SBP >=140 mmHg or mean DBP >=90 mmHg, regardless of diagnosis or medication use.
Clean fuel	Electricity, LPG, natural gas, or biogas.
Biomass/solid fuel	Coal/lignite, charcoal, wood, straw/shrubs/grass, agricultural crop residues, or dung.
Excluded fuel categories	Kerosene, other fuel, no cooking at home, no response, and missing fuel.
Sampling design	PSU and stratum identifiers made unique by year, pooled weight, and centered single-unit strata.
Continuous systolic blood pressure	Mean systolic blood pressure in mmHg from valid DHS measurements, analyzed as a continuous outcome in supplementary linear models.
Continuous diastolic blood pressure	Mean diastolic blood pressure in mmHg from valid DHS measurements, analyzed as a continuous outcome in supplementary linear models.
Body mass index	BMI calculated from DHS anthropometric measurements and reported as kg/m2; added as a continuous anthropometric characteristic and as a covariate in the extended adjustment sensitivity model.
Employment status	Self-reported employment status harmonized across DHS rounds and included only in the extended adjustment sensitivity model.
Current smoking status	Self-reported current smoking status harmonized across DHS rounds and included only in the extended adjustment sensitivity model.

Operational definitions correspond to the analytic dataset derived from pooled DHS Peru 2014–2024.
