# Supplementary material for: Biomass Cooking Fuel Use and Screen-Detected Hypertension Among Peruvian Women Without Known Hypertension: A National Cross-Sectional Study
Source: Life (Basel). 2026 Aug 6;16(8):1293. doi: 10.3390/life16081293 (PMC13514650; doi:10.3390/life16081293)
Supplement: Supplementary file 1 [file life-16-01293-s001.zip › BiomassHTN_SM_Table_S3_IncludedExcluded.pdf]

Supplementary Table S3. Comparison between included and excluded women.

Variable	Total	Excluded	Included	ASD
Age, years	30.7 (9.8)	31.9 (9.9)	30.6 (9.8)	0.133
Cooking fuel				
Clean fuel	82.6	86.5	82.3	0.117
Biomass/solid fuel	17.4	13.5	17.7	0.117
Area of residence				
Urban	81.2	86.0	80.9	0.14
Rural	18.8	14.0	19.1	0.14
Natural region				
Metropolitan Lima	36.3	40.3	36.0	0.088
Rest of coast	26.6	26.7	26.6	0.001
Highlands	24.2	20.2	24.5	0.104
Jungle	12.9	12.8	12.9	0.001
Wealth quintile				
Poorest	16.5	11.6	16.9	0.152
Poor	20.3	15.7	20.7	0.13
Middle	21.6	21.0	21.6	0.014
Rich	21.7	24.1	21.5	0.061
Richest	19.9	27.7	19.4	0.197
Known hypertension				
No known HTN	92.9	0.0	100.0	0.0
Known HTN	7.1	100.0	0.0	0.0
Screen-detected hypertension				
No	89.7	61.8	91.9	0.761
Yes	10.3	38.2	8.1	0.761

ASD: absolute standardized difference between groups. ASD greater than 0.1 indicates relevant imbalance.
