# Supplementary material for: Biomass Cooking Fuel Use and Screen-Detected Hypertension Among Peruvian Women Without Known Hypertension: A National Cross-Sectional Study
Source: Life (Basel). 2026 Aug 6;16(8):1293. doi: 10.3390/life16081293 (PMC13514650; doi:10.3390/life16081293)
Supplement: Supplementary file 1 [file life-16-01293-s001.zip › BiomassHTN_SM_Table_S4_KnownVsUnknown.pdf]

Supplementary Table S4. Characteristics of women with known HTN versus women without known HTN.

Variable	Total	No known HTN	Known HTN	ASD
Age, years	30.7 (9.8)	30.6 (9.8)	31.9 (9.9)	0.133
Cooking fuel				
Clean fuel	82.6	82.3	86.6	0.119
Biomass/solid fuel	17.4	17.7	13.4	0.119
Area of residence				
Urban	81.2	80.9	86.1	0.141
Rural	18.8	19.1	13.9	0.141
Natural region				
Metropolitan Lima	36.3	36.0	40.3	0.088
Rest of coast	26.6	26.6	26.8	0.003
Highlands	24.2	24.5	20.1	0.106
Jungle	12.9	12.9	12.9	0.0
Wealth quintile				
Poorest	16.5	16.9	11.5	0.153
Poor	20.3	20.7	15.7	0.131
Middle	21.6	21.6	21.0	0.015
Rich	21.7	21.5	24.1	0.062
Richest	19.9	19.4	27.7	0.198
Measured elevated blood pressure				
No	89.7	91.9	61.6	0.767
Yes	10.3	8.1	38.4	0.767

HTN: hypertension; ASD: absolute standardized difference between groups. The total sample includes nonpregnant women aged 15–49 years with valid blood pressure measurements and complete covariates, regardless of self-reported diagnosis.
