# Supplementary material for: Biomass Cooking Fuel Use and Screen-Detected Hypertension Among Peruvian Women Without Known Hypertension: A National Cross-Sectional Study
Source: Life (Basel). 2026 Aug 6;16(8):1293. doi: 10.3390/life16081293 (PMC13514650; doi:10.3390/life16081293)
Supplement: Supplementary file 1 [file life-16-01293-s001.zip › BiomassHTN_SM_Table_S5_FullModel.pdf]

Supplementary Table S5. Fully adjusted model for the main analysis.

Term	Adjusted PR (95% CI)	p
Clean fuel	1.00 (reference)	
Biomass/solid fuel	1.20 (1.10-1.31)	<0.001
Age, spline 1	1.04 (1.02-1.05)	<0.001
Age, spline 2	0.92 (0.88-0.97)	<0.001
Age, spline 3	1.25 (1.10-1.41)	<0.001
Altitude, spline 1	1.00 (1.00-1.00)	<0.001
Altitude, spline 2	1.02 (1.01-1.03)	<0.001
Altitude, spline 3	0.98 (0.97-0.98)	<0.001
Urban (reference)	1.00 (reference)	
Rural	0.86 (0.79-0.94)	<0.001
Metropolitan Lima (reference)	1.00 (reference)	
Rest of coast	0.80 (0.75-0.86)	<0.001
Highlands	0.62 (0.52-0.74)	<0.001
Jungle	0.64 (0.59-0.70)	<0.001
No education (reference)	1.00 (reference)	
Primary	0.81 (0.70-0.93)	0.003
Secondary	0.82 (0.71-0.94)	0.005
Higher education	0.83 (0.71-0.96)	0.015
Poorest (reference)	1.00 (reference)	
Poor	1.05 (0.95-1.16)	0.322
Middle	1.13 (1.01-1.27)	0.038
Rich	1.21 (1.07-1.37)	0.002
Richest	1.26 (1.11-1.44)	<0.001
DHS 2014 (reference)	1.00 (reference)	
DHS 2015	0.82 (0.72-0.93)	0.002
DHS 2016	0.88 (0.77-1.01)	0.069
DHS 2017	0.93 (0.81-1.06)	0.281
DHS 2018	1.07 (0.95-1.22)	0.258
DHS 2019	0.92 (0.81-1.06)	0.260
DHS 2020	1.10 (0.95-1.26)	0.203
DHS 2021	1.29 (1.14-1.46)	<0.001
DHS 2022	1.14 (1.00-1.29)	0.048
DHS 2023	0.84 (0.73-0.96)	0.010
DHS 2024	0.92 (0.80-1.04)	0.191

PR: prevalence ratio; CI: confidence interval. The model includes age and altitude using restricted cubic splines with four knots; categorical covariates are listed by level. The reference category for each variable is the baseline category included in the main analysis.
