# Supplementary material for: Biomass Cooking Fuel Use and Screen-Detected Hypertension Among Peruvian Women Without Known Hypertension: A National Cross-Sectional Study
Source: Life (Basel). 2026 Aug 6;16(8):1293. doi: 10.3390/life16081293 (PMC13514650; doi:10.3390/life16081293)
Supplement: Supplementary file 1 [file life-16-01293-s001.zip › BiomassHTN_SM_Table_S6_Sensitivity_REVISED_HIGHLIGHTED.pdf]

Supplementary Table S6. Sensitivity analyses by outcome definition and analytical population.

| Analysis | n | Events | Adjusted PR (95% CI) | p |
|---|---|---|---|---|
| Main: women without known HTN; outcome by measured BP | 301,575 | 18,571 | 1.20 (1.10-1.31) | <0.001 |
| All women; total HTN definition | 321,184 | 38,180 | 1.18 (1.11-1.25) | <0.001 |
| All women; measured BP only | 321,362 | 24,707 | 1.18 (1.09-1.27) | <0.001 |
| Excluding medicated women only | 309,994 | 20,233 | 1.20 (1.10-1.31) | <0.001 |
| Excluding previous diagnosis only | 301,575 | 18,571 | 1.20 (1.10-1.31) | <0.001 |
| Restricted to age >=18 years | 273,165 | 17,105 | 1.22 (1.12-1.33) | <0.001 |
| Including pregnant women | 312,187 | 19,100 | 1.19 (1.09-1.30) | <0.001 |
| Excluding Metropolitan Lima | 263,833 | 15,111 | 1.19 (1.09-1.30) | <0.001 |
| Common support of the propensity score | 288,326 | 17,333 | 1.20 (1.10-1.32) | <0.001 |
| Extended adjustment: main model + BMI, employment status, and current smoking status | 293,404 | 18,045 | 1.23 (1.12-1.34) | <0.001 |

PR: prevalence ratio; CI: confidence interval; HTN: hypertension.
