# Supplementary material for: Biomass Cooking Fuel Use and Screen-Detected Hypertension Among Peruvian Women Without Known Hypertension: A National Cross-Sectional Study
Source: Life (Basel). 2026 Aug 6;16(8):1293. doi: 10.3390/life16081293 (PMC13514650; doi:10.3390/life16081293)
Supplement: Supplementary file 1 [file life-16-01293-s001.zip › BiomassHTN_SM_Table_S7_FuelSpecific_REVISED_HIGHLIGHTED.pdf]

Supplementary Table S7. Analysis by specific fuel type.

Exposure	Unweighted n	Weighted prevalence of HTN, %	Crude PR (95% CI)	Adjusted PR (95% CI)
Clean fuel	223,076	8.5	1.00	1.00
Wood	71,142	6.5	0.77 (0.72-0.82)	1.25 (1.14-1.36)
Coal/charcoal	2,612	7.0	0.82 (0.62-1.07)	1.00 (0.76-1.31)
Other solid fuels	4,745	3.8	0.44 (0.35-0.55)	0.92 (0.72-1.17)

PR: prevalence ratio; CI: confidence interval; HTN: hypertension. The reference category is clean fuel. Adjusted models include age, altitude, area of residence, natural region, education, wealth quintile, and survey year.
