# Supplementary material for: Biomass Cooking Fuel Use and Screen-Detected Hypertension Among Peruvian Women Without Known Hypertension: A National Cross-Sectional Study
Source: Life (Basel). 2026 Aug 6;16(8):1293. doi: 10.3390/life16081293 (PMC13514650; doi:10.3390/life16081293)
Supplement: Supplementary file 1 [file life-16-01293-s001.zip › BiomassHTN_SM_Table_S8_EffectMod_REVISED_HIGHLIGHTED.pdf]

Supplementary Table S8. Effect modification.

Modifier	Stratum	n	Events	n exposed	Adjusted PR (95% CI)	p	interaction p value
Area of residence	Urban	210,481	14,005	17,532	1.28 (1.14-1.43)	<0.001	0.005
Area of residence	Rural	91,094	4,566	60,967	1.09 (0.96-1.24)	0.189	0.005
Natural region	Metropolitan Lima	37,742	3,460	194	1.09 (0.55-2.14)	0.811	0.015
Natural region	Rest of coast	91,323	6,418	7,211	1.39 (1.20-1.62)	<0.001	0.015
Natural region	Highlands	100,337	5,293	48,306	1.08 (0.95-1.24)	0.237	0.015
Natural region	Jungle	72,173	3,400	22,788	1.12 (0.97-1.28)	0.132	0.015
Altitude	<1500 m	192,937	12,688	27,104	1.27 (1.13-1.44)	<0.001	0.291
Altitude	1500-2499 m	24,418	1,616	7,941	1.08 (0.85-1.37)	0.539	0.291
Altitude	>=2500 m	84,220	4,267	43,454	1.12 (0.97-1.29)	0.139	0.291
Age group	15-24	91,150	4,803	24,794	1.27 (1.09-1.48)	0.002	0.477
Age group	25-34	104,443	5,884	23,971	1.37 (1.20-1.57)	<0.001	0.477
Age group	35-49	105,982	7,884	29,734	1.05 (0.94-1.18)	0.346	0.477
DHS period	2014-2017	110,051	6,374	29,090	1.40 (1.19-1.64)	<0.001	<0.001
DHS period	2018-2020	79,039	5,077	19,357	1.00 (0.86-1.17)	0.959	<0.001
DHS period	2021-2024	112,485	7,120	30,052	1.12 (0.97-1.29)	0.116	<0.001
Wealth quintile	Poorest	80,022	4,015	61,664	1.25 (1.10-1.43)	<0.001	<0.001
Wealth quintile	Poor	77,152	4,192	14,797	1.16 (1.02-1.32)	0.025	<0.001
Wealth quintile	Middle	61,796	3,996	1,865	1.11 (0.83-1.47)	0.487	<0.001
Wealth quintile	Rich	48,471	3,521	166	1.28 (0.47-3.46)	0.625	<0.001
Wealth quintile	Richest	34,134	2,847	7	NE (sparse exposed stratum)		<0.001

PR: prevalence ratio; CI: confidence interval; HTN: hypertension. Stratum-specific models use the same main adjustment set: age, altitude, area of residence, natural region, education, wealth quintile, and survey year, excluding the stratifying variable when appropriate.
