# Supplementary material for: Biomass Cooking Fuel Use and Screen-Detected Hypertension Among Peruvian Women Without Known Hypertension: A National Cross-Sectional Study
Source: Life (Basel). 2026 Aug 6;16(8):1293. doi: 10.3390/life16081293 (PMC13514650; doi:10.3390/life16081293)
Supplement: Supplementary file 1 [file life-16-01293-s001.zip › BiomassHTN_SM_Table_S9_Positivity.pdf]

Supplementary Table S9. Positivity diagnostics.

Diagnostic	Value
Lower limit of common support	0.0001
Upper limit of common support	0.9648
Propensity score range: clean fuel	0.0000-0.9648
Outside common support: clean fuel	13,154 / 223,076 (5.9%)
Propensity score range: biomass/solid fuel	0.0001-0.9696
Outside common support: biomass/solid fuel	95 / 78,499 (0.1%)
Exposure x region x area x wealth cells	66
Sparse cells, n < 30	8
Sparse exposed cells, n < 30	8
n exposed in the richest quintile	7

Diagnostics based on the propensity score for biomass/solid fuel use estimated with weighted logistic regression and the same covariates as in the main model.
